# Translation of hyperpolarized [^13^C,^15^N_2_]urea MRI for novel human brain perfusion studies

**DOI:** 10.1038/s44303-025-00073-3

**Published:** 2025-03-20

**Authors:** Yaewon Kim, Hsin-Yu Chen, Tanner Nickles, Illia Shkliar, Duy Dang, James Slater, Charlie Wang, Jeremy W. Gordon, Chou T. Tan, Chris Suszczynski, Sri Maddali, Adam Gaunt, Rui Chen, Javier Villanueva-Meyer, Duan Xu, Peder E. Z. Larson, John Kurhanewicz, Robert A. Bok, Susan Chang, Daniel B. Vigneron

**Affiliations:** 1 Department of Radiology and Biomedical Imaging, University of California, San Francisco, CA USA; 2 ISOTEC Stable Isotope Division, MilliporeSigma, Merck KGaA, Miamisburg, OH USA; 3 General Electric Healthcare, Niskayuna, NY USA; 4 Department of Neurological Surgery, University of California, San Francisco, CA USA

**Keywords:** Biomedical engineering, Medical imaging

## Abstract

This study developed a new approach to produce sterile, hyperpolarized [^13^C,^15^N_2_]urea as a novel molecular imaging probe and applied it for first-ever healthy brain volunteer studies. Hyperpolarized [^13^C,^15^N_2_]urea, as a small, metabolically inert molecule, offers significant advantages for perfusion imaging due to its endogenous nature and excellent safety profile. The developed methods achieved a hyperpolarized [^13^C,^15^N_2_]urea solution (132 ± 6 mM) with 27.4 ± 5.6% polarization and a T_1_ = 50.4 ± 0.2 s. In healthy brain volunteer studies, high-resolution ^13^C imaging captured blood flow with a spatial resolution of 7.76 × 7.76 × 15 (or 10) mm^3^ over ~1 min following hyperpolarized [^13^C,^15^N_2_]urea injection, visualizing detailed vascular structures. Time-to-peak and centroid analyses showed consistent arterial and venous signal patterns across subjects. Findings suggest hyperpolarized [^13^C,^15^N_2_]urea may have applications beyond brain imaging, including the non-invasive perfusion assessment in various organs, cancer microenvironment, and renal function, paving the way for clinical translation.

## Introduction

Over the past decade, hyperpolarized (HP) carbon-13 MRI, utilizing dissolution dynamic nuclear polarization (dDNP)^1^, has become a powerful clinical-research molecular imaging approach^2–4^. This stable-isotope (non-radioactive) technology enables real-time imaging of cellular metabolic processes by tracking the uptake and enzymatic conversions of injected HP substrates, most commonly [1-^13^C]pyruvate, to provide unique insights into abnormal metabolism in disease states^5–9^. While HP ^13^C MRI has primarily been employed to investigate metabolic pathways, its ability to capture dynamic blood flow measurements has also positioned it as a powerful tool for perfusion imaging, particularly in the brain^10^.

Perfusion imaging is essential for diagnosing and monitoring neurological conditions like stroke, brain tumors, and neurodegenerative diseases, offering critical insights into blood flow and tissue health. Among HP ^13^C agents explored for this purpose, ^13^C-urea has gained significant attention in preclinical studies due to its unique properties as an endogenous, metabolically inert molecule that directly reflects blood flow and tissue perfusion^11–13^. Compared to Gadolinium (Gd)-based MR contrast agents, urea’s lower molecular weight—approximately 15 times smaller—allows for rapid distribution into extracellular, interstitial spaces and microvasculature, making it uniquely effective in characterizing tissue perfusion^12,13^. In addition, urea offers a direct proportionality of signal-to-probe concentration and an inherently high contrast-to-noise ratio due to the absence of background signal^14^, whereas Gd contrast agents in ^1^H DCE-MRI often suffer from non-specific uptake, non-linear relationship between signal intensity and concentration, and reduced sensitivity in non-enhancing tumors^15^.

The dual-labeled [^13^C,^15^N_2_]urea provides much longer relaxation times (T_2_ increase by 250-fold^15^) than using natural abundance ^14^N and, therefore, greatly benefits higher usable polarizations and longer lifetimes for HP ^13^C MRI^16,17^. Preclinical research has validated the use of HP [^13^C,^15^N_2_]urea for safe and effective investigation of conditions such as tumor hypoxia^18,19^, cardiomyopathy^20^, renal ischemia^21–23^, and acute kidney injury^24,25^ by observing decreased urea perfusion with high SNR. While it perfuses into most tissues, urea typically does not cross the blood–brain barrier (BBB)^26^ and, in fact, has been used clinically as a hyperosmolar agent to decrease brain swelling^27^. Also, ex vivo brain tissue studies from patients with Alzheimer’s, Huntington’s, Parkinson’s disease, Lewy Body, and vascular dementia demonstrated elevated cerebral urea^28,29^.

For human research, HP [^13^C,^15^N_2_]urea was first translated as part of a dual-agent probe with [1-^13^C]pyruvate, enabling simultaneous measurement of pyruvate metabolism and urea tissue perfusion^30,31^. In a first-in-human study involving a prostate cancer patient, the co-administration of 150 mM HP [1-^13^C]pyruvate and 35 mM [^13^C,^15^N_2_]urea revealed a mismatch between metabolism and tissue-perfusion that is characteristic of high-grade prostate cancer, where decreased urea tissue perfusion aligned with increased pyruvate metabolism^32^. This finding underscored the potential of HP [^13^C,^15^N_2_]urea to provide unique insights into tumor perfusion and hypoxia in aggressive pathologies. Furthermore, this may eventually serve as a potential method to investigate blood–brain barrier integrity in conditions such as dementia, trauma, other neurodegenerative disorders, and aging.

In the dual-agent probe, the pyruvate-to-urea concentration ratio was 4:1, resulting in a urea probe concentration of ~35 mM^30^. Compared to the coarser resolution achieved in the dual-agent study for tissue perfusion and tumor hypoxia, this project developed a single-agent urea probe at a 4-fold higher concentration, enabling higher resolution imaging of brain vasculature and perfusion. To achieve this, we designed a standard operating procedure (SOP) for producing sterile HP single-agent [^13^C,^15^N_2_]urea and obtained IRB & FDA-IND approvals. This protocol was validated for clinical translation and feasibility studies of urea perfusion, regional vasculature, and its distribution in the healthy human brain.

## Results

### Standard operating procedure (SOP) development for sterile HP urea production

In this project, we successfully developed and optimized a new SOP for the on-site production of sterile hyperpolarized (HP) urea, following Good Manufacturing Practice (GMP) guidelines as outlined in the U.S. Code of Federal Regulation Title 21, Part 212. To dissolve solid urea and achieve a DNP-compatible sample with ~9 M urea concentration, lactic acid was used as a solvent due to its similar physical properties to pyruvic acid (i.e., a self-glassing agent) as previously demonstrated^33^. The urea/lactic acid/electron paramagnetic agent (EPA) mixture, sterile water for injection (SWFI) for dissolution, and buffer for neutralization were prepared as described in the Methods section and loaded into a GE Pharmacy Kit. The composition of the Pharmacy Kit preparation for the HP [^13^C,^15^N_2_]urea probe is summarized in Table 1.

**Table 1:** Pharmacy kit (fluid path) chemical preparations for sterile hyperpolarized [^13^C,^15^N_2_]urea probe

Components	Chemical composition	Function
Cryovial	1.524 ± 0.01 g GMP [^13^C,^15^N_2_]urea in lactic acid (food grade 88%)*	Perfusion-sensing probe
	*Mixed with 12 mM AH11501 sodium salt	Electron paramagnetic agent
Dissolution syringe	41.0 ± 0.05 g SWFI	Heated and pressurized to dissolve frozen imaging probe in the cryovial
Receiver bag	21.45 ± 0.05 g SWFI 8.4 ± 0.05 g Tris/EDTA/NaOH buffer	Diluent Neutralize lactic acid

*EDTA* ethylenediaminetetraacetic acid, *NaOH* sodium hydroxide, *SWFI* sterile water for injection, *Tris* Tris(hydroxyethyl)aminomethane.

**Fig. 1:**
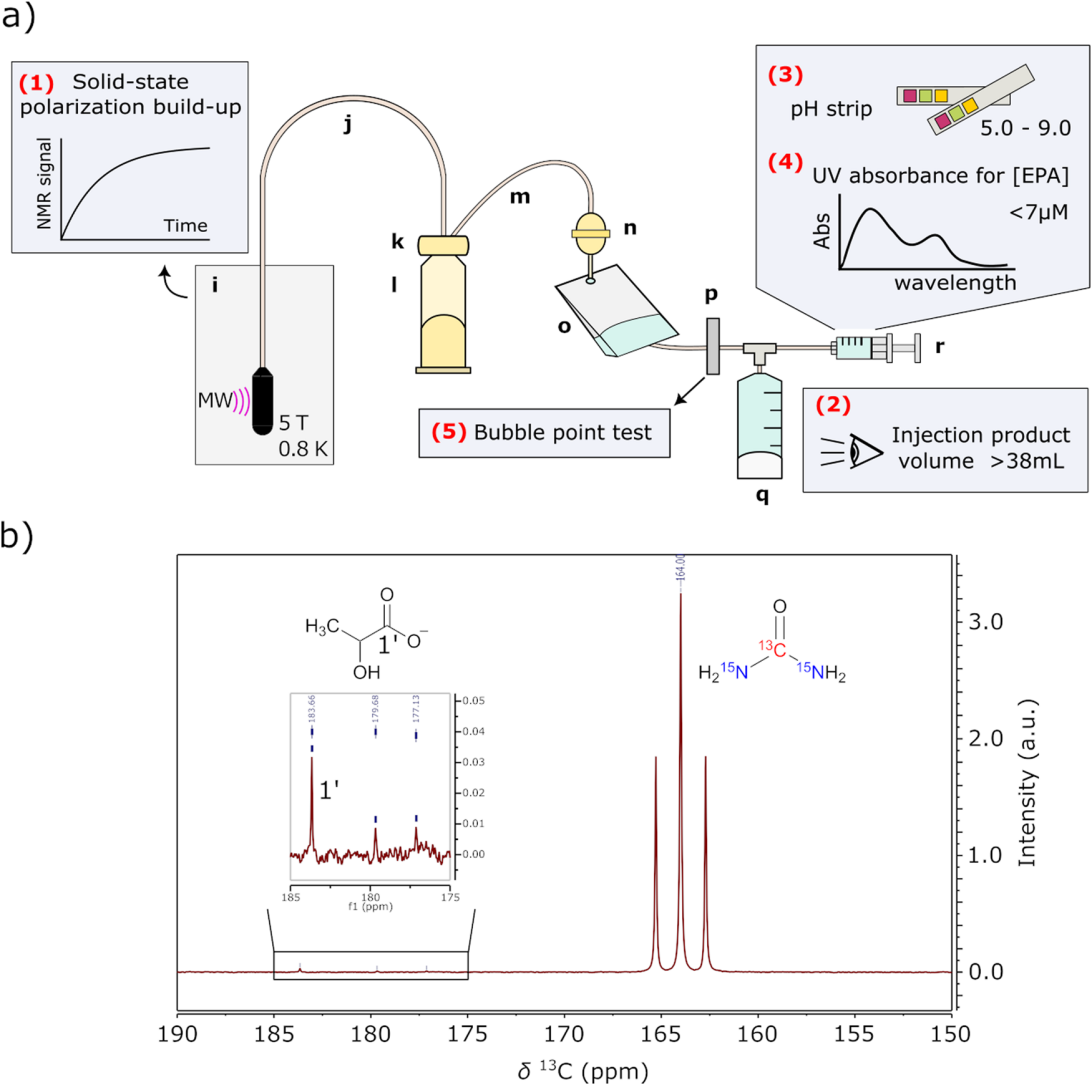
Fluid path workflow and quality control tests for preparing hyperpolarized [^13^C,^15^N_2_]urea. **a** Fluid path (Pharmacy Kit) components and steps for pre-release quality control tests: (i) Cryovial in the SPINlab system at 0.8 K with microwave irradiation; (j) Dual-lumen dissolution tubing; (k) Dissolution syringe valve; (l) Dissolution syringe; (m) Single-lumen transfer tubing; (n) electron paramagnetic agent (EPA) filter; (o) Receiver bag; (p) sterility filter; (q) MedRad® syringe for patient administration; (r) quality control (QC) syringe; (1) Solid-state ^13^C NMR measurements performed on the frozen urea sample to monitor polarization build-up (2) Visual inspection of injection product volume (3) pH test of the sample collected in the QC syringe (4) EPA concentration measurements by UV spectrophotometry (5) Bubble point test to ensure the sterility filter integrity. **b** First timepoint HP ^13^C NMR spectrum of the injection product acquired on a 1.4 T benchtop NMR spectrometer.

Figure 1a illustrates the components of the GE Pharmacy Kit, including a cryovial with the urea/lactic acid/EPA solution (i), transfer tubing (j, m), a dissolution valve (k), a dissolution syringe filled with 41 ml SWFI (l), a size-exclusion EPA filter (n), a receiver bag containing SWFI and NaOH-Tris buffer (o), a terminal sterilization filter (p), a power-injector syringe (Medrad, Bayer Germany) (q), and a QC syringe (r).

In addition, Fig. 1a outlines the quality control (QC) measurements required before releasing the HP urea probe, which are also summarized in Table S1. Firstly, solid-state ^13^C NMR signals from the sample in a cryovial were monitored during microwave irradiation to confirm a more than 100-fold increase in signal intensity, ensuring that there would be sufficient hyperpolarization before dissolution (Fig. 1a(1)). During the dissolution process in the SPINlab DNP polarizer, the HP urea was rapidly dissolved by superheated SWFI from the dissolution syringe and transferred through tubing to a receiver bag, passing through a size-exclusion filter that removed EPA. In the receiver bag, the dissolved product was neutralized with Tris-NaOH, resulting in an HP urea solution at physiological temperature and pH, referred to as the “injection product.” The injection product then passed through a terminal sterilization filter before reaching the administering syringe and QC syringe. Upon completion of the dissolution process, the injection product’s volume was visually inspected to confirm that more than 38 mL was collected in the Medrad syringe (Fig. 1a(2)). The Medrad syringe was then prepared for patient administration. Meanwhile, 3–4 mL of the injection product in the QC syringe underwent pH testing (acceptable range: 5.0–9.0) using a pH strip and EPA concentration testing (less than 7 μM) using a UV spectrophotometer (Fig. 1a(3), (4)). Finally, a bubble point test was performed to ensure the sterility filter’s integrity (Fig. 1a(5)). Once all five QC tests were successfully passed, the pharmacist released the injection product for human administration. Post-dissolution QC tests, including pH electrode measurement, endotoxin, and sterility testing, were also conducted. The SOP was validated through three consecutive successful Process Qualification trials (Table S2).

### Imaging probe characterization

After 3.5 h of polarization, the solid-state ^13^C polarization of [^13^C,^15^N_2_]urea reached ~90% of the maximum achievable polarization determined from the fitting curve, with a build-up time constant of 5480 s (Fig. S1). Following rapid dissolution, a HP [^13^C,^15^N_2_]urea solution with a neutral pH of 7–8 was successfully prepared. A representative HP ^13^C NMR spectrum of the injection product displays the urea signal as a triplet, attributed to J-coupling between ^13^C and ^15^N spins (20.2 Hz) at 164 ppm, along with a lactate signal at natural abundance at 183.7 ppm (Fig. 1b). In addition, two minor peaks observed upfield from the lactate signal at 176.1 and 178.8 ppm were identified as lactate dimers. No other impurities were detected in the spectrum.

Thermal ^13^C NMR spectrum analysis revealed that the urea concentration was 132 ± 6 mM, nearly four times higher than the urea concentrations used in the prior co-polarized pyruvate and urea experiments (~35 mM^30^), while the natural-abundance lactate concentration was 81 ± 9 mM. The liquid-state ^13^C polarization of urea at the time of dissolution was calculated to be 27.4 ± 5.6%. The average concentration of the residual EPA was measured to satisfy specifications at 4.1 ± 2.2 μM, with a pH of 8.2 ± 0.2. The T_1_ of the HP ^13^C urea was 43.3 ± 2.6 s at 1.4 T on the benchtop NMR and 50.4 ± 0.2 s at 3 T MRI.

### First-in-human brain imaging with HP [^13^C,^15^N_2_]urea

**Fig. 2:**
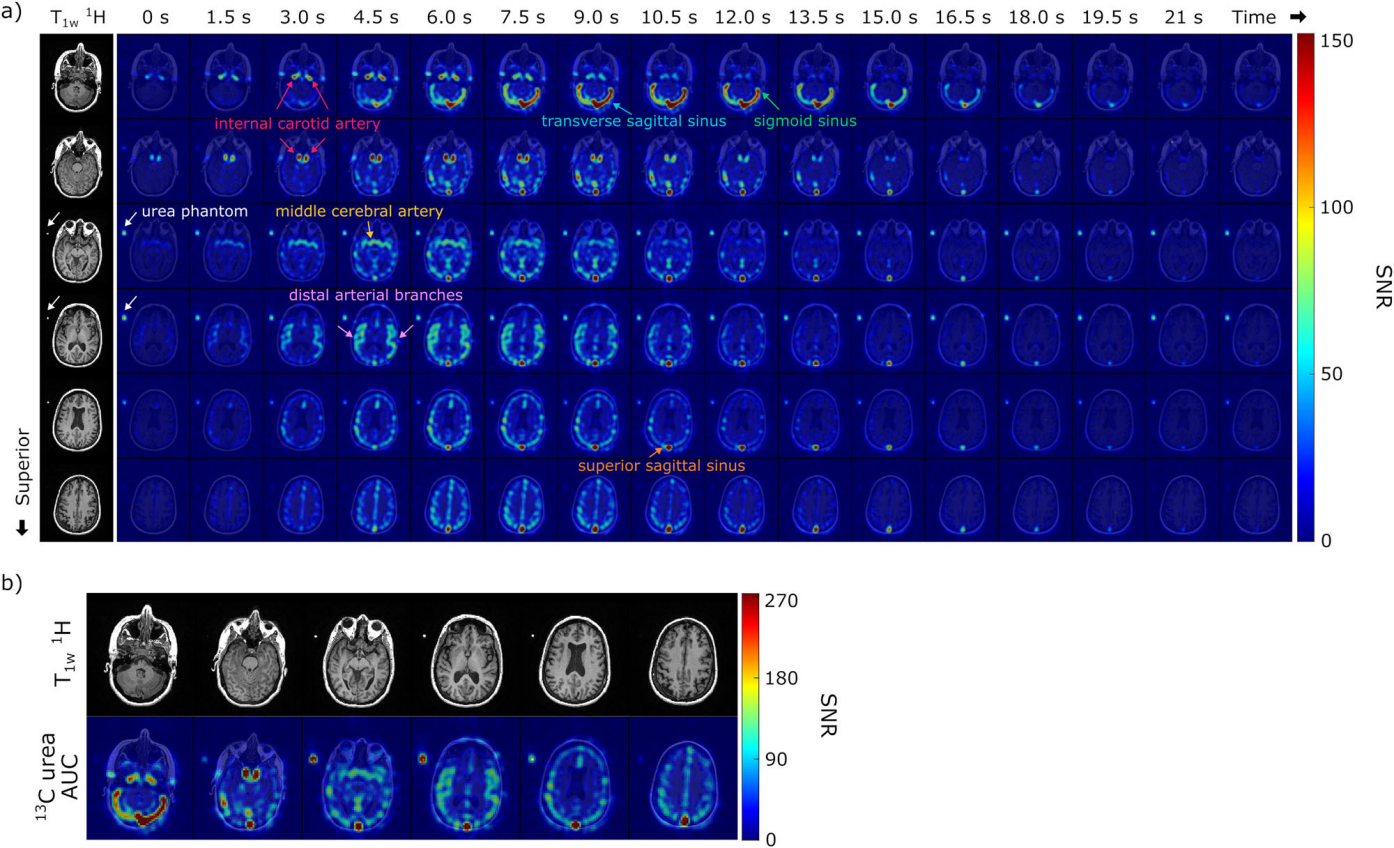
Representative hyperpolarized [^13^C,^15^N_2_]urea images from a healthy brain volunteer. **a** Corresponding ^1^H anatomical images and the dynamic HP [^13^C,^15^N_2_]urea images (7.76 × 7.76 × 15 mm^3^) overlaid on the ^1^H anatomical images are displayed. A total of 20 slices and 32 timeframes were acquired, and this figure displays the middle 6 slices and the first 16 timeframes (1.5 s temporal resolution). White arrows indicate the signal from the urea phantom. **b** Total signal (area-under-curve, AUC) images of urea from the same subject. The urea images overlaid on the corresponding ^1^H anatomical images are shown.

The time course of HP [^13^C,^15^N_2_]urea signals from healthy brain volunteers is illustrated in Fig. 2a and Supporting Information Figs. S2 and S3. The spatial resolution achieved was 7.76 × 7.76 × 15 mm^3^, comparable to the highest reported resolution of HP [1-^13^C]pyruvate acquisition (7.5 × 7.5 × 15 mm^3^)^34^. Urea images clearly delineated the cerebrovascular system, including the anterior circulation (internal carotid and middle cerebral arteries), posterior circulation (vertebral and basilar arteries), and dural venous sinuses (transverse/superior sagittal sinus, sigmoid sinus). These images captured dynamic signal intensity changes corresponding to the in-flow and washout of HP [^13^C,^15^N_2_]urea compounded with T_1_ relaxations of HP magnetization.

Initially, the urea bolus signal appeared in the internal carotid and middle cerebral arteries, followed by the distal arterial branches, and eventually in the venous system, specifically well seen in the transverse and sagittal sinuses (Fig. 2a). Asymmetric flow of urea into the left and right transverse sinuses was observed in most volunteer datasets, consistent with normal anatomic variation^35^. The peak dynamic SNR in this dataset was 380, observed within the confluence of sinuses at the 8th timepoint (10.5 s after the saline injection ended). An average peak SNR of 253 ± 136 was measured in the superior sagittal sinus across all volunteer datasets (*n* = 8) with one exception where the peak signal was observed in the superficial temporal artery. Area-under-curve (AUC) images from a representative subject, shown in Fig. 2b, highlighted signals from the blood vessels, but no urea signal was observed in the brain parenchyma, consistent with urea not crossing the blood–brain barrier (BBB).

**Fig. 3:**
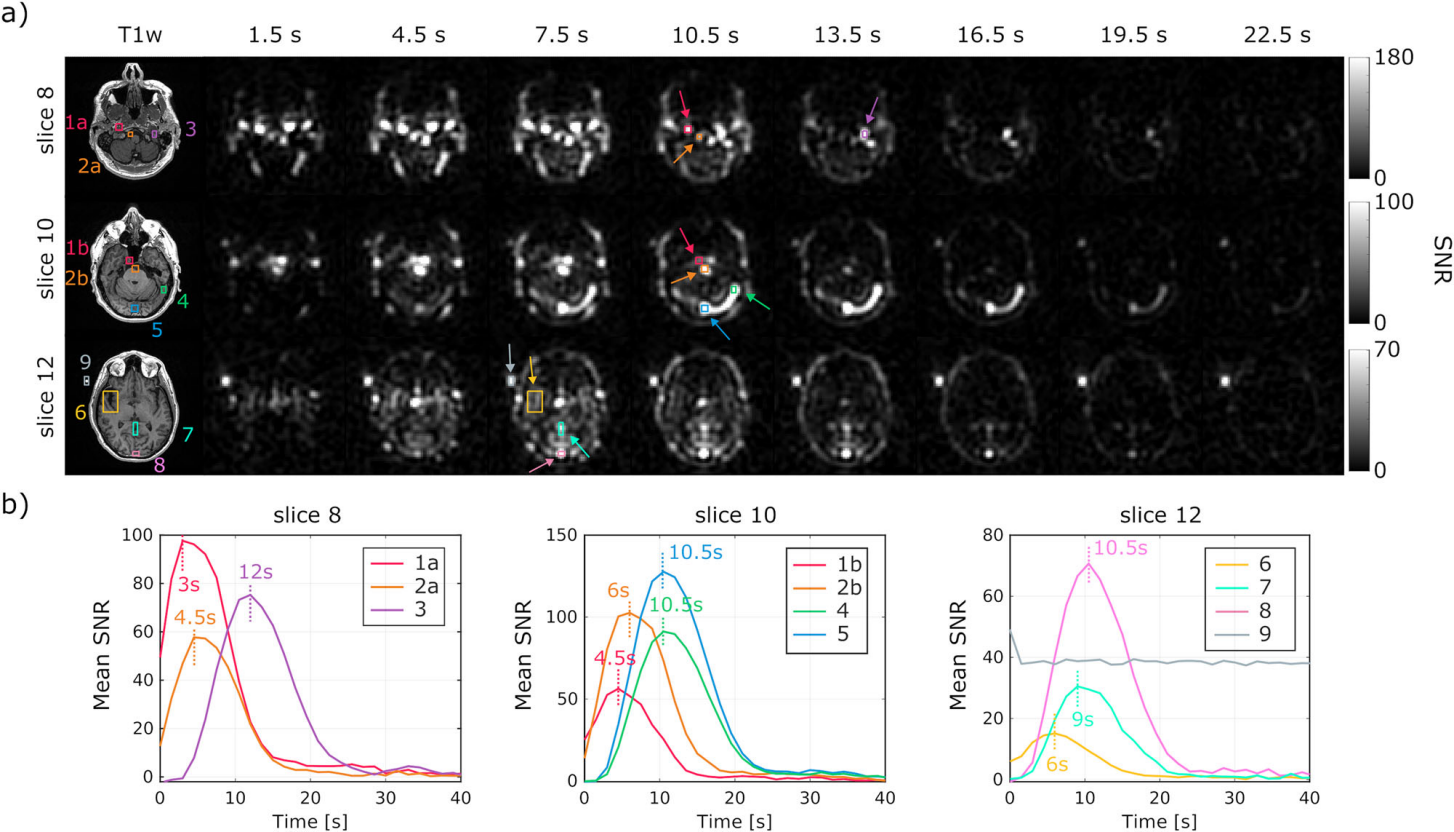
Time-course analysis of hyperpolarized [^13^C,^15^N_2_]urea signals in a healthy brain volunteer. **a** Dynamic HP [^13^C,^15^N_2_]urea images of selected slices from a volunteer in grayscale. Every other timeframe between 1.5 and 22.5 s is shown. Eleven ROIs are indicated by numbers (1–9) and letters (used for the same anatomical structures but on different slices) on the ^1^H images, and by arrows on the ^13^C images (1: internal carotid artery; 2: basilar artery; 3: sigmoid sinus/internal jugular vein; 4: transverse sinus; 5: confluence of sinuses; 6: M2 middle cerebral artery; 7: vein of Galen; 8: superior sagittal sinus; 9: urea phantom). **b** Time-course plots of urea signals from the ROIs, with time-to-peak points indicated by dotted vertical lines.

Figure 3b displays dynamic urea images from selected slices of another volunteer. The time course of urea signals from regions of interest (ROIs) placed on arteries and veins across different axial slices revealed that arterial signal peaks occurred 4.5 to 9 s earlier than venous signals. As expected, larger differences were observed in the inferior slices (ex. slice 8 vs slice 12). With a temporal resolution of 1.5 s, we detected a slight difference in peak times between the urea signals in the internal carotid (1) and basilar arteries (2), with a delay of one timeframe. Across four datasets with a temporal resolution of 1.5 s, the average peak time difference between urea signals in the internal carotid artery and venous sinuses on the same axial slice was 6.1 ± 1.4 s (*n* = 4).

**Fig. 4:**
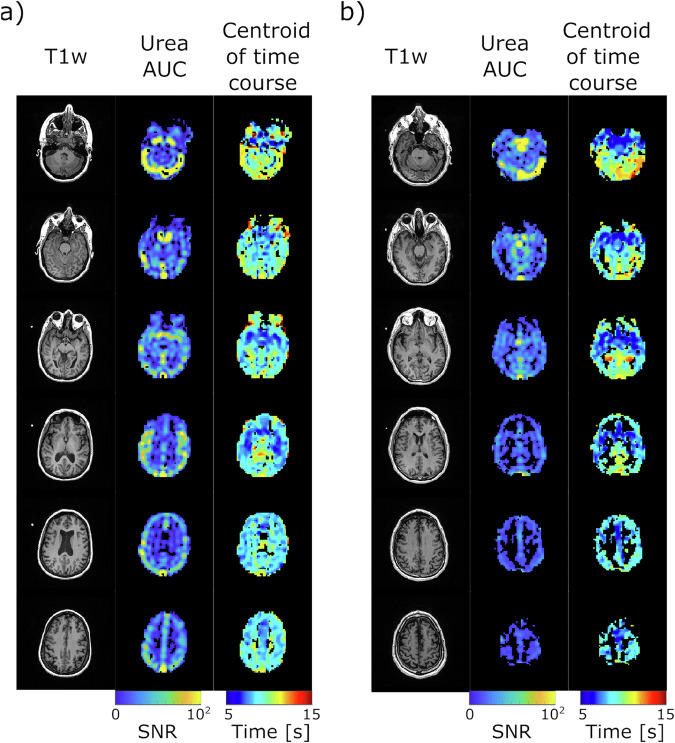
Hyperpolarized [^13^C,^15^N_2_]urea area-under-curve (AUC) and time-course centroid maps from two subjects. ^1^H anatomical images (first column), urea AUC images (second column), and time course centroid maps third column) acquired from two subjects (**a**, **b**). The HP urea AUC and time course centroid maps were masked for voxels with SNR greater than 10 in the hyperpolarized urea AUC images.

The time course centroid maps represent the center of mass of the time course^36^, providing insights into the distribution of urea signals over time across the brain. As shown in Fig. 4, the centroid maps from two representative subjects demonstrate a symmetric distribution of urea signals between the left and right hemispheres. Signals from the arteries appear more “blue”, indicating an earlier centroid, while venous signals are more “red” in these images, reflecting a delayed centroid. Gaussian distribution analysis revealed two distinct time regimes within the centroid maps: for the dataset in Fig. 4a, the mean and standard deviation (μ, σ) were (8.8, 1.2) seconds and (10.4, 2.6) seconds, while for the dataset in Fig. 4b, they were (7.5, 0.9) seconds and (9.4, 1.4) seconds (Fig. S4). The distributions with smaller mean values likely represent arterial signals, while those with larger mean values correspond to venous signals, indicating a difference of 1.6 and 1.9 s between the two regimes. Across four datasets collected with the same temporal resolution (1.5 s), an average difference of 2.0 ± 0.3 s was observed between arterial and venous portions of the signals. This consistency suggests the reproducibility of the measurement across individuals.

**Fig. 5:**
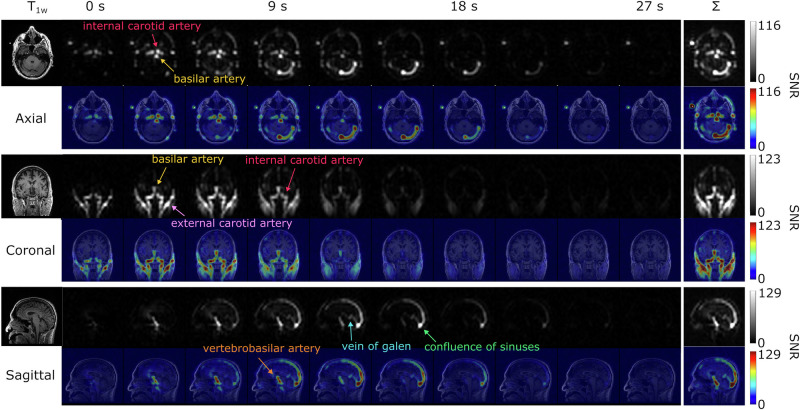
Dynamic hyperpolarized [^13^C,^15^N_2_]urea images in multiple planes. Dynamic HP [^13^C,^15^N_2_]urea images (7.76 × 7.76 × 10 mm^3^) and corresponding AUC images (Σ) acquired from a volunteer are displayed in axial (top), coronal (middle), and sagittal (bottom) planes.

In a separate study, a slice thickness of 10 mm was used while maintaining the same in-plane resolution. Representative dynamic urea images are displayed in Fig. 5, featuring axial, coronal, and sagittal planes. In the axial images, clear signals from the sigmoid sinus and the confluence of sinuses were visible. The coronal images depicted the urea bolus flowing through the internal carotid artery, while sagittal images showed the urea signal first appearing in the basilar artery, then moving through the superior sagittal sinus to the confluence of sinuses.

## Discussion

This study was the first to successfully prepare sterile, single agent hyperpolarized (HP) [^13^C,^15^N_2_]urea and demonstrate its feasibility and safety for human use. None of the four volunteers experienced any adverse effects during or after the urea injections, including when administered twice in a single exam. The urea concentration used in this imaging study was sub-physiological, as urea naturally occurs in the body at millimolar concentrations, reaching over a hundred millimolar in the renal medulla. The administered dose of 5.6 mg/kg was ~2–5 times lower than normal blood urea levels (naturally produced as a waste product of metabolism; 10.5–30 mg/kg) and 150 to 300 times lower than the urea doses clinically used for conditions like glaucoma^37^, cerebral edema^38,39^, and hyponatremia^40^, which range from 1 to 1.5 g/kg. In addition, a previous study investigating the toxicity of [^13^C,^15^N_2_]urea in Sprague Dawley rats, co-administered with hyperpolarized [1-^13^C]pyruvate, found no adverse effects on vital signs, laboratory results, or the gross pathology of major organs^30^. Based on the successful process qualification tests performed using the SOP developed in this study, our HP [^13^C,^15^N_2_]urea protocol received both FDA IND (under IND# 109956) and UCSF IRB approval for studies in healthy volunteers.

For this project, lactic acid was selected as the solvent and glassing agent for dissolving solid urea due to its self-glassing properties at low temperatures, as demonstrated in a previous study where neat lactic acid was successfully hyperpolarized without additional glassing agents^33^. As a naturally occurring metabolite, lactate minimizes the risk of adverse reactions, and its lower reactivity compared to pyruvate reduces the chance of side reactions with urea, as confirmed by NMR analyses. Its endogenous nature ensures biological compatibility for clinical applications. Lactic acid also offers practical advantages: It is less viscous than glycerol, which has been used as a glassing agent for urea in preclinical studies^12–14^, facilitating easier handling. In this study, food-grade lactic acid (88%), compliant with United States Pharmacopeia (USP) standards for purity and safety for consumption, was used. The 12% water aided the dissolution of urea to ~9 M, ensuring a uniform and concentrated solution. This material is also inexpensive and readily available, further supporting its practical use.

However, lactic acid’s lower acidity (pKa = 3.86) compared to pyruvic acid (pKa = 2.50) decreased the efficiency of radical removal via size-exclusion filters, as the EPA precipitates more effectively in more acidic environments. To counter this, we reduced the initial EPA concentration to 12 mM (down from the typical 15 mM used in pyruvate studies) and adjusted the release criterion for EPA concentration from 5 μM to 7 μM. These modifications improved the success rate in meeting the quality control standards by consistently lowering the final EPA concentrations to below 7 μM. While the reduced EPA concentration led to a slight increase in the polarization build-up time constant to ~5500 s (~1.3 times longer than with 15 mM EPA), it remained within a practical range for clinical applications. Future improvements may include the use of C_18_ cartridges for more effective EPA removal, which could support higher EPA concentrations and shorter polarization build-up times while maintaining residual EPA under the specified limit.

The significant advantages of [^13^C,^15^N_2_]urea over [^13^C]urea with natural abundance ^14^N for clinical translation have been described in the preclinical literature^16,20^, including its much longer T_1_ and T_2_ relaxation times, which provide higher in vivo signals for perfusion imaging particularly when using bSSFP-based acquisition. The longer T_1_ relaxation time of [^13^C,^15^N_2_]urea also makes it less sensitive to low magnetic fields encountered during clinical quality-control tests and sample transfer, preserving hyperpolarization, which begins to decay immediately after dissolution due to T_1_ relaxation. As demonstrated in preclinical studies, replacing the current dissolution solvent (H_2_O) with deuterated water (D_2_O) is expected to further increase both T_1_ and T_2_ relaxation times^41^, potentially enhancing SNR in future applications.

With the current SOP, our imaging results demonstrated that single-agent HP [^13^C,^15^N_2_]urea MRI can dynamically assess cerebral perfusion with high sensitivity, offering novel flow and perfusion information in addition to what gadolinium-based contrast agents provide. Although urea does not normally cross the blood–brain barrier, this characteristic makes it a potentially valuable tool for evaluating blood–brain barrier integrity in conditions such as dementia, multiple sclerosis, traumatic brain injury, and brain tumors that show increased permeability due to blood–brain barrier leakage^42^. Compared to conventional MRI techniques for measuring cerebral blood flow such as Arterial Spin Labeling (ASL)^43^, HP [^13^C,^15^N_2_]urea MRI offers the benefit of positive contrast with no background signal and much longer T_1_ relaxation times, providing more sensitive detection of perfusion abnormalities from arteries to capillaries to veins. Beyond brain applications, HP [^13^C,^15^N_2_]urea MRI may be beneficial for non-invasive assessment of tissue perfusion, cancer aggressiveness, cardiac perfusion, urea transporters, kidney function, liver function, and other perfusion-related diseases.

In this study, we developed and optimized a new approach for producing sterile, hyperpolarized [^13^C,^15^N_2_]urea as a new MR molecular imaging agent, and successfully demonstrated its feasibility and safety in imaging the cerebral vasculature and blood flow in healthy volunteers. The results demonstrated that HP [^13^C,^15^N_2_]urea could be a viable and safe MR imaging probe, with significant potential for perfusion imaging in the brain. HP urea effectively captures blood flow by detecting signals from both arterial and venous circulation, making it a promising tool for assessing changes in blood–brain barrier integrity in neurological disorders, as well as evaluating perfusion characteristics in various other organs. This study provides a foundation for the clinical translation of HP urea, offering new possibilities for non-invasive perfusion imaging across multiple organ systems.

## Methods

### Preparation of HP [^13^C,^15^N_2_]urea probe for clinical studies

To produce a single dose of HP urea probe, 810 mg of [^13^C,^15^N_2_]urea (GMP grade; ISOTEC, MilliporeSigma, Miamisburg, OH) was dissolved with 1.39 g of natural abundance (not C-13 enriched) lactic acid (Food grade 88%; Lab Alley, Spicewood, TX), and 28.5 mg of AH111501 (GE Healthcare; an electron paramagnetic agent (EPA)), was added to 1.905 g of the [^13^C,^15^N_2_]urea/lactic acid mixture. This resulted in a solution of ~9.0 M [^13^C,^15^N_2_]urea with 12 mM EPA.

For hyperpolarization, 1.52 g of the prepared [^13^C,^15^N_2_]urea solution was loaded into a cryovial and polarized on a 5 T SPINlab system (GE Healthcare) operating at 0.8 K. The sample was polarized for 3–4 h with microwaves at a frequency of 139.88 GHz, which was 0.01 GHz lower than the microwave frequency used for polarizing pyruvate. Following polarization, the frozen urea sample was rapidly dissolved with 41 g of super-heated (130 °C), pressurized sterile water for injection (SWFI) and subsequently neutralized with 29.85 g of 600 mM sodium hydroxide (NaOH) and 333 mM Tris(hydroxyethyl)aminomethane (Tris) buffer.

### Nuclear magnetic resonance (NMR) spectroscopy

To characterize T_1_ and polarization of the HP urea imaging probe, HP ^13^C NMR spectra were acquired on a 1.4 T benchtop NMR system (Magritek) with the following parameters: 1° RF flip angle, 5 s temporal resolution for a total of 128 repetitions. After the HP ^13^C magnetization was fully relaxed, the signal intensity at thermal equilibrium was measured by applying 1% v/v Gd-DTPA (Magnevist®, Bayer, Whippany, NJ) and using parameters including a 10 s temporal resolution, a 90° flip angle, and 512 averages. The T_1_ for [^13^C,^15^N_2_]urea was determined from an exponential fit to the dynamic HP signal intensities after correcting for the signal loss caused by RF pulses. Liquid-state polarization of the HP urea was calculated by comparing the urea signal from the first HP spectrum to the thermal-equilibrium spectrum, then back-calculating to the time of dissolution using T_1_ measured from the HP NMR data. A 9.4 T NMR instrument (Bruker Avance III) was used to determine the urea concentration of the dose for injection.

### HP ^13^C MRI

The HP ^13^C urea data of the brain collected in this study were acquired from four healthy volunteers (Median age 37, range 26–64, 3 males, 1 female) using a 3D balanced steady-state free precession (bSSFP) sequence with a stack-of-spiral acquisition^31^ following the injection of HP [^13^C,^15^N_2_]urea. The 3D-bSSFP pulse sequence consisted of a series of excitation pulses (50°) with alternating phases, employing 6 interleaves and 20 (or 30) stacks for k_z_ encoding, with 8-ms TR and a field-of-view (FOV) of 45.0 × 45.0 × 30.0 cm^3^ with a matrix size of 58 × 58 × 20 (or 30). At the beginning of each timeframe, four catalyzation pulses with increasing amplitudes (i.e., 6°, 19°, 34.5°, and 46.5°) were applied, and the reversed order of catalyzation pulses was used at the end of the timeframe to restore the magnetization back to the z-axis. A spoiler gradient was added to crush residual transverse magnetization. A non-selective 1-ms RF pulse was used for excitation. The acquisition achieved a spatial resolution of 7.76 × 7.76 × 15 mm^3^ with a temporal resolution of 1.5 s (*N* = 4) or 3 s (*N* = 2) or a spatial resolution of 7.76 × 7.76 × 10 mm^3^ with a temporal resolution of 1.5 s (*N* = 2) over thirty-two timeframes. For anatomic reference, proton T_2_-weighted and 3D T_1_-weighted images were acquired. All experiments were performed on a 3 T clinical MR scanner (Premier, GE Healthcare, Waukesha, WI). A ^1^H/^13^C dual-frequency transmit/receive radiofrequency (RF) coil with volume ^13^C transmit and multichannel 8 ^1^H & 24 ^13^C channel receive (Rapid Biomedical, Rimper, Germany) was used in this study. Transmit RF power was calibrated on a head-shaped phantom containing unenriched ethylene glycol (HOCH_2_CH_2_OH, anhydrous, 99.8%, Sigma Aldrich, St. Louis, MO) prior to the volunteer study. All human studies were conducted with informed consent following a protocol approved by both the Institutional Review Board (IRB) and the Food and Drug Administration for an investigational new drug (FDA IND).

### HP ^13^C data processing and analysis

HP ^13^C urea data were reconstructed by first gridding the k-space data using the Kaiser-Bessel gridding method^44^, followed by an inverse Fourier transform to generate the reconstructed images. The resulting images were then pre-whitened, coil-combined^45^, and zero-padded to match the resolution of the proton anatomical images. For time-to-peak analysis, regions of interest (ROIs) were drawn over different branches of arteries and veins, and the signal intensities within the ROIs were averaged to plot the dynamic curves. The centroid of the urea dynamic curve was calculated as the time-weighted average of signal intensity, obtained by dividing the sum of time-weighted signal intensities by the total area under the curve^36^. In addition, two Gaussian functions were fitted to histograms for analyzing the time course centroids. All processing and analysis were performed using MATLAB.

## Supplementary information

FirstUrea_brainMRI_SI_revision_final_2025

## Data Availability

Deidentified data will be made available upon request to the Authors, after the initiation of formal data sharing agreement as required by the data sharing policy at our institution (UCSF).
