# Supplementary material for: Translation of hyperpolarized [13C,15N2]urea MRI for novel human brain perfusion studies
Source: Npj Imaging. 2025 Mar 20;3:11. doi: 10.1038/s44303-025-00073-3 (PMC11925798; doi:10.1038/s44303-025-00073-3)
Supplement: Supplementary file 1 — FirstUrea_brainMRI_SI_revision_final_2025 [file 44303_2025_73_MOESM1_ESM.docx]

Supporting information for “Translation of Hyperpolarized [^13^C,^15^N_2_]Urea MRI for Novel Human Brain Perfusion Studies”

Yaewon Kim^1^, Hsin-Yu Chen^1^, Tanner Nickles^1^, Illia Shkliar^1^, Duy Dang^1^, James Slater^1^, Charlie Wang^1^, Jeremy W. Gordon^1^, Chou T. Tan^2^, Chris Suszczynski^2^, Sri Maddali^2^, Adam Gaunt^3^, Rui Chen^3^, Javier Villanueva-Meyer^1^, Duan Xu^1^, Peder E.Z. Larson^1^, John Kurhanewicz^1^, Robert A. Bok^1^, Susan Chang^4^, Daniel B. Vigneron^1,4^

^1^Department of Radiology and Biomedical Imaging, University of California, San Francisco, CA, USA.

2ISOTEC Stable Isotope Division, MilliporeSigma, Merck KGaA, Miamisburg, OH, USA.

^3^General Electric Healthcare, Niskayuna, New York, USA.

^4^Department of Neurological Surgery, University of California, San Francisco, CA, USA.

Corresponding author: Yaewon Kim ([yaewon.kim@ucsf.edu](mailto:dan.vigneron@ucsf.edu))

| **Test** | **Analytical Procedure** | **Acceptance Criteria** |
| --- | --- | --- |
| Residual AH111501 concentration | UV Absorbance | < 7μM |
| pH | pH Strip | 5.0 – 9.0 |
| Drug product volume | Visual Inspection | >38 ml |
| Filter Integrity | Bubble point test | Manufacturer specification |
| ^13^C Nuclear Polarization | Solid State NMR | >100x increase |

Table S1: Quality Control (QC) tests for hyperpolarized [^13^C,^15^N_2_]urea injection product

| **Trial** | **Urea concentration (mM)** | **AH111501 concentration (μM)** | **pH** | **Injection Product Volume** | **Filter Integrity** |
| --- | --- | --- | --- | --- | --- |
| 1 | 126.8 | 4.9 | 8.3 | >38ml | Passed |
| 2 | 134.1 | 6.2 | 8.1 | >38ml | Passed |
| 3 | 143.8 | 6.1 | 8.0 | >38ml | Passed |

Table S2. Process Qualification (PQ) trials to validate the standard operating procedure (SOP) prior to FDA IND submission (that was approved before human studies).


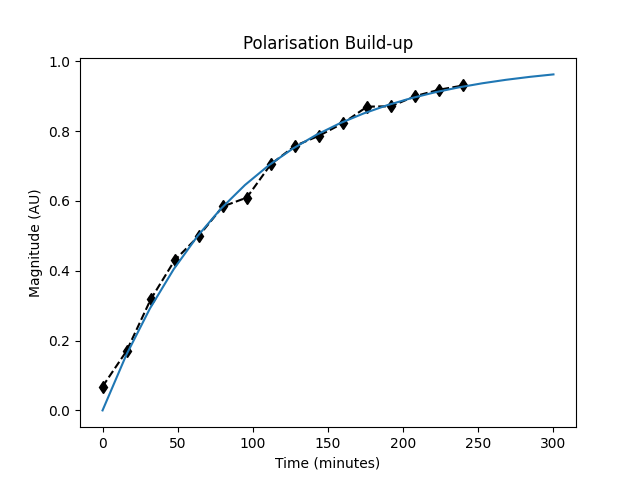


Figure S1.Polarization build-up of [^13^C,^15^N_2_]urea measured at 0.8 K and 5T. The blue solid line represents the fitted curve based on the equation (S[t] = S[0]x(1-exp(t/T_1,build-up_)+intercept). The polarization build-up time constant (T_1,build-up_) was determined to be 91.3 minutes (5480 sec).


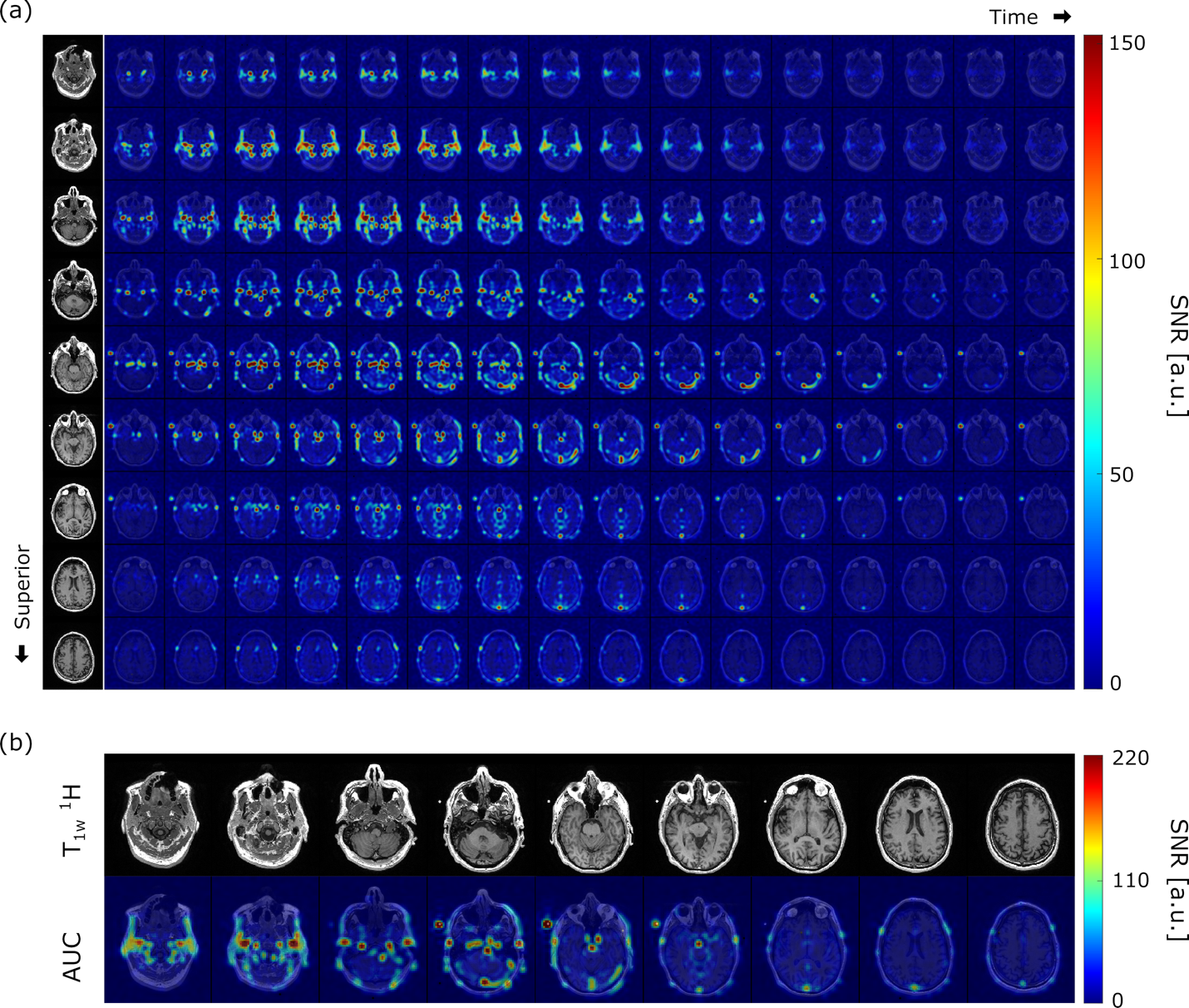


Figure S2. Hyperpolarized [^13^C,^15^N_2_]urea images from a healthy brain volunteer (#3). (a) The corresponding ^1^H anatomical images and the dynamic 7.76 x 7.76 x 15 mm^3^ [^13^C,^15^N_2_]urea images overlaid on the ^1^H anatomical images are displayed. Twenty total slices and thirty-two timeframes were acquired, and the middle 9 slices and the first 16 timeframes are shown here. (b) Area-under-curve (AUC) images of urea from the same subject. The urea images overlaid on the corresponding ^1^H anatomical images are shown.


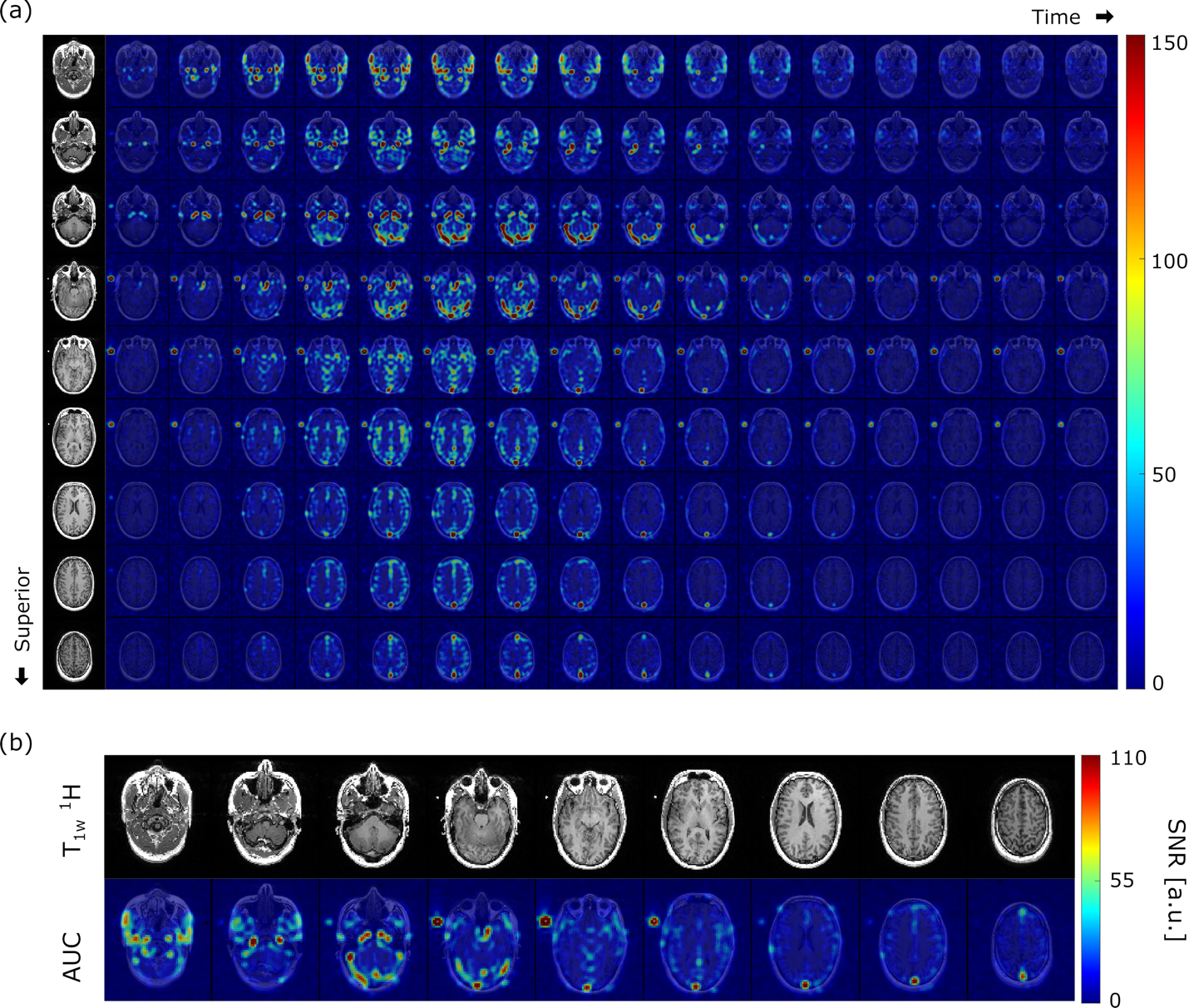


Figure S3. Hyperpolarized [^13^C,^15^N_2_]urea images from a healthy brain volunteer (#4). (a) The corresponding ^1^H anatomical images and the dynamic 7.76 x 7.76 x 15 mm^3^ [^13^C,^15^N_2_]urea images overlaid on the ^1^H anatomical images are displayed. Twenty total slices and thirty-two timeframes were acquired, and the middle 9 slices and the first 16 timeframes are shown here. (b) Area-under-curve (AUC) images of urea from the same subject. The urea images overlaid on the corresponding ^1^H anatomical images are shown.


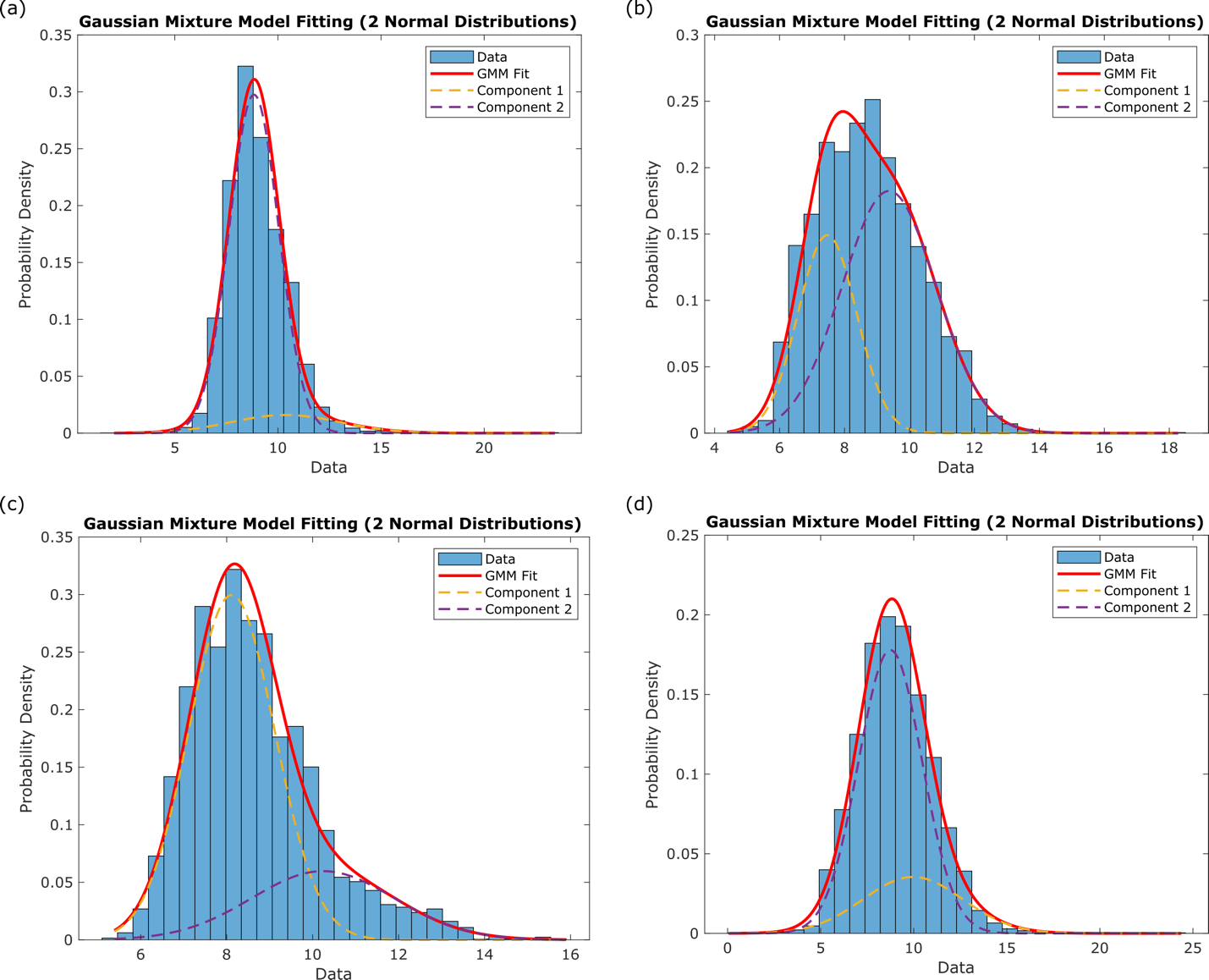


Figure S4. Histograms of time course centroid distributions from four volunteer datasets. Data were fitted with two Gaussian components, shown by dashed yellow and purple curves. The sum of the two components is shown by a red solid curve. The mean (μ) and standard deviation (σ) values for each component were as follows: (a) component 1: (8.8, 1.2), component 2: (10.4, 2.6); (b) component 1: (7.5, 0.9), component 2: (9.4, 1.4); (c) component 1: (8.1, 1.0), component 2: (10.2, 1.7); (d) component 1: ((7.5, 1.3), component 2: (9.8, 1.8). The datasets were acquired with a spatial resolution of 7.76 x 7.76 x 15 mm^3^ and a temporal resolution of 1.5 sec.
